# School achievement in adolescence and the risk of mental disorders in early adulthood: a Finnish nationwide register study

**DOI:** 10.1038/s41380-023-02081-4

**Published:** 2023-05-02

**Authors:** Tarja Weckström, Marko Elovainio, Laura Pulkki-Råback, Kimmo Suokas, Kaisla Komulainen, Sari Mullola, Petri Böckerman, Christian Hakulinen

**Affiliations:** 1 Department of Psychology and Logopedics, Faculty of Medicine, University of Helsinki, Helsinki, Finland; 2 Research Program Unit, Faculty of Medicine, University of Helsinki, Helsinki, Finland; 3 Finnish Institute for Health and Welfare, Helsinki, Finland; 4 Faculty of Social Sciences, Tampere University, Tampere, Finland; 5 Department of Education, University of Helsinki, Helsinki, Finland; 6 Teachers College Columbia University, National Center for Children and Families (NCCF), New York, NY USA; 7 School of Business and Economics, University of Jyväskylä, Jyväskylä, Finland; 8 Labour Institute for Economic Research LABORE, Helsinki, Finland; 9 IZA Institute of Labor Economics, Bonn, Germany

**Keywords:** Schizophrenia, Depression, Psychology

## Abstract

School grades in adolescence have been linked to later psychiatric outcomes, but large-scale nationwide studies across the spectrum of mental disorders are scarce. In the present study, we examined the risk of a wide array of mental disorders in adulthood, as well as the risk of comorbidity, associated with school achievement in adolescence. We used population-based cohort data comprising all individuals born in Finland over the period 1980–2000 (*N* = 1,070,880) who were followed from age 15 or 16 until a diagnosis of mental disorder, emigration, death, or December 2017, whichever came first. Final grade average from comprehensive school was the exposure, and the first diagnosed mental disorder in a secondary healthcare setting was the outcome. The risks were assessed with Cox proportional hazards models, stratified Cox proportional hazard models within strata of full-siblings, and multinomial regression models. The cumulative incidence of mental disorders was estimated using competing risks regression. Better school achievement was associated with a smaller risk of all subsequent mental disorders and comorbidity, except for eating disorders, where better school achievement was associated with a higher risk. The largest associations were observed between school achievement and substance use disorders. Overall, individuals with school achievement more than two standard deviations below average had an absolute risk of 39.6% of a later mental disorder diagnosis. By contrast, for individuals with school achievement more than two standard deviations above average, the absolute risk of a later mental disorder diagnosis was 15.7%. The results show that the largest mental health burden accumulates among those with the poorest school achievement in adolescence.

## Introduction

Students who perform well in school, i.e., have good school achievement, make an easier transition into adulthood, obtain a higher level of education, and are more likely to achieve occupational and economic success and maintain better health [1–3]. Approximately half of all mental health problems manifest in adolescence [4], coinciding with the time of secondary schooling. From public health perspective, it is crucial to identify factors from the psychosocial environment that can inform about the distribution of psychiatric risk from adolescence into adulthood. School grades are objective measures of academic achievement that are independent of memory or interpretation biases. Because school grades have been associated with mental disorders [5], they may help with such identification.

In epidemiological studies, poor school achievement, measured by school grades, has been associated with a greater risk of developing many adverse psychiatric outcomes in adolescence and adulthood, such as schizophrenia [6], bipolar disorder [7, 8], depression [8–11], substance use [12], and suicide [13, 14]. To date, large-scale longitudinal prospective evidence has focused mostly on specific mental disorders, as only one Swedish national study has investigated the associations between school achievement and a range of major mental disorders [5]. Moreover, the association between school achievement and comorbidity, a central aspect of mental health [15] which implies more complex problems and worse overall health outcomes, has not been investigated previously.

Using high-quality population-based Finnish administrative records comprising over 1.1 million persons, we examined the association between school achievement and the risk of a wide array of subsequent mental disorders as well as the risk of their comorbidity. In addition to reporting relative risks, we calculated absolute risks, i.e., cumulative incidences, which are more informative for mental health service planning, policy makers, and the general public than relative risk estimates. Utilizing comprehensive national health and other administrative records, we were also able to account for a variety of potential individual and environmental confounders, such as urbanicity [16], family socioeconomic characteristics [17], and parental mental illness [18, 19], which are known to be associated with school achievement and mental health outcomes. Lastly, we also conducted the analyses using a within-sibling research design to account for otherwise unobserved shared family characteristics that affect both school achievement and the incidence of mental disorders.

## Methods

### Study population

Several national registers containing demographic, health, and educational information were linked using the unique identification number assigned to all Finnish citizens. The study population consisted of persons born in Finland between 1980 and 1999 and who had two Finnish parents. Exclusion criteria from the sample were death, emigration, or a mental disorder diagnosis prior to the follow-up period. In the original data, 2.3% of the study population (*N* = 26,795) had missing data on education. The missing data could imply school drop-out, but also schooling abroad, as well as serious somatic illness, mental disability, or other significant barriers to finishing compulsory school. Due to the heterogeneity of the group without register-based educational information, we excluded them from the analyses. The remaining 1,070,880 individuals formed our final analytic sample and were followed from 1 August in the year they graduated from the nine-year compulsory education (around the age of 15 or 16) until the first diagnosed mental disorder, death, emigration, or the end of follow-up on 31 December 2017, whichever occurred first. Thus, those born in 1980 were followed from 1 August 1996 until 31 December 2017, and those born in 1999 were followed from 1 August 2015 until 31 December 2017 at the latest. The ethics committee of the National Institute of Health and Welfare (THL/22/6.02.01/2019) approved the study. Data were linked with the permission of the Statistics Finland (TK-53-1696-16) and the National Institute of Health and Welfare.

### School achievement

In Finland, education is free-of-charge from preschool to higher academic education. At the age of seven, children begin a nine-year compulsory comprehensive school, where the curriculum and grading are based on national guidelines that all schools are obliged to follow. After the ninth grade, children apply to secondary education such as a three-year general upper secondary education or vocational education and training.

The school achievement measure was the mean of the final ninth year grades from comprehensive school. The original grades were on a scale ranging from 4 to 10, where higher numbers indicate better school achievement (4 = fail, 5–6 = poor, 7–8 = good, 9–10 = excellent). The mean was standardized using a z-score transformation (mean 0, SD 1), and school achievement was also analyzed as a five-level categorical variable: −2SD (≤−2SD); −1SD (>−2SD – ≤−1SD); 0 (>−1SD – <1SD); 1SD (≥1SD – <2SD); 2SD (≥2SD).

The grades were obtained from the National Joint Application Register containing the information on school grades of students in the final year of comprehensive school between 1996 to 2015. Information on school grades from the years 1996–2007 was available only for those who applied for secondary education. From 2008 to 2015, information on school grades was available for all students in the final year of compulsory school attendance, including those who did not apply for secondary education. The school achievement measure was calculated based on all subjects including compulsory (e.g., mother tongue and literature (Finnish or Swedish), the first foreign language, mathematics, history and social studies, religion/ethics, physical education, mathematics, music, and visual arts) and optional (e.g., the second foreign language) subjects. To account for variation in the data collection method and possible differences in grading between years, the school achievement measures were standardized within each year of graduation.

### Mental disorders

Diagnoses of mental disorders were obtained from the Finnish National Hospital Discharge Register, which contains data on virtually all inpatient visits since 1970 and outpatient visits in special healthcare since 1998 and includes the ICD-10 diagnostic classification (or previous editions of the manual according to the year of visit) for the reason for each visit.

The first inpatient or outpatient secondary care contacts with any mental disorder (ICD-10 diagnoses F00–F99) was the main outcome. In addition, the following eight diagnostic sub-categories were analyzed: 1) mental and behavioral disorders due to psychoactive substance use (F10–F19), 2) schizophrenia spectrum disorders including schizophrenia, schizotypal, and delusional disorders (F20–F29), 3) bipolar disorder (F30–F31), 4) depression (F32–F33), 5) neurotic, stress-related, and somatoform disorders (F40–F48), 6) eating disorders (F50–F50.9), 7) nonorganic sleep disorders (F51), and 8) disorders of adult personality and behavior (F60–F69).

### Covariates

Demographic, socioeconomic, and intergenerational covariates included in the analyses were sex (0 = male, 1 = female), birth year, degree of urbanicity in residential location (0 = missing, 1 = urban, 2 = semi-urban, 3 = rural), parental education level at the time of their child’s graduation (0 = missing, 1 = comprehensive, 2 = upper secondary, 3 = lower tertiary, 4 = Bachelor’s or equivalent, 5 = Master’s or higher), parental income level in quintiles at time of their child’s graduation (calculated in relation to the study population) (0 = missing, 1 = 1^st^ quintile, 2 = 2^nd^ quintile, 3 = 3^rd^ quintile, 4 = 4^th^ quintile, 5 = 5^th^ quintile), and parental mental health history (0=no psychiatric diagnosis, 1=any psychiatric diagnosis before the follow-up period). Sample characteristics are described in Supplementary Table S1.

### Statistical analysis

The study population was followed from 1^st^ August in the year that they graduated (around age 16) until the first psychiatric diagnosis, death, emigration, or the end of follow-up on 31^th^ December 2017, whichever came first. We used Cox proportional hazard models to estimate the association between school grades at the age of 16 with the later risk of first diagnosed mental disorder. Model 1 evaluated the associations while adjusting for sex, the year of birth and time-varying calendar year period as covariates; Model 2 was adjusted for all covariates, i.e., sex, the year of birth, time-varying calendar year period, parental education level, parental income level, parental mental health history, and urbanicity; Model 3 was adjusted for all covariates and it was estimated using stratified Cox proportional hazard models conducted within strata of full-sibling sets, which enabled us to account for otherwise unobserved confounding by shared family characteristics that could affect both school achievement and the incidence of mental disorders [20]. We used the standardized school grade mean (>−1SD – <1SD) as the reference group for the estimation of the hazard ratios (HRs).

The cumulative incidences were estimated using competing risks regression [19], based on Fine and Gray’s proportional subhazards model, treating death and emigration as competing events. Psychiatric comorbidity was calculated as the count of separate mental disorders with which a person was diagnosed during the follow-up period. The associations of school achievement with comorbidity were assessed with multinomial logistic regression, using those without any mental disorders as the reference category. The analyses were conducted between January 2022 and March 2023, using Stata version 16.1 [21].

## Results

Over 12.2 million years of total analysis time at risk and under observation, 144,324 persons (13.5%) were diagnosed with any mental disorder. Table 1 shows the incidence rates of mental disorders in the sample, and the hazard ratios with 95% CIs for the associations of continuous school achievement with psychiatric diagnoses. The incidence rate for any mental disorder diagnosis was 3.25 per 100,000 person-years at risk. Model 1 shows the HRs for the associations of school achievement with mental disorders while adjusting for sex, the year of birth, and time-varying calendar year period. Although several other confounders, including parental income and education level, parental mental health, and urbanicity, were accounted for in Model 2, the estimated HRs differed only slightly from the HRs of Model 1. In both models, the hazard ratios were all below 1, indicating that better school achievement was associated with a smaller risk of a subsequent mental disorder, except for eating disorders; in Model 2, where all covariates were included, each improved standardized school grade was associated with a 25% greater risk of a subsequent eating disorder (HR = 1.25, 95% CI 1.21–1.29). In the sibling analyses accounting for otherwise unobserved shared family characteristics, the associations were slightly attenuated, and the confidence intervals were wider (Model 3). Analyses stratified by sex suggested that there were only marginal differences between males and females in the association of school achievement with mental disorders (Supplementary Table S2).

**Table 1:** Descriptive information with incidence rates of the mental disorder diagnoses examined, and hazard ratios (HRs) with 95% confidence intervals (CIs) for the associations of standardized school achievement in adolescence with later mental disorder diagnoses.

Mental disorder	Number of diagnoses	Incidence rate	Model 1		Model 2		Model 3	
			HR	95% CI	HR	95% CI	HR	95% CI
Any	144,324	1186	0.70	0.70–0.70	0.71	0.71–0.72	0.71	0.70–0.72
Substance use	26,605	204	0.47	0.47–0.48	0.49	0.48–0.50	0.54	0.52–0.56
Schizophrenia spectrum	14,230	108	0.75	0.73–0.76	0.73	0.72–0.75	0.70	0.68–0.73
Bipolar	8863	67	0.77	0.75–0.79	0.78	0.76–0.80	0.78	0.74–0.82
Depression	67,954	533	0.74	0.73–0.74	0.76	0.75–0.76	0.75	0.73–0.76
Anxiety	69,969	549	0.70	0.70–0.71	0.71	0.71–0.72	0.72	0.70–0.73
Eating	7341	56	1.32	1.29–1.36	1.25	1.21–1.29	1.14	1.06–1.23
Sleep	4466	34	0.67	0.65–0.69	0.68	0.66–0.71	0.71	0.71–0.71
Personality	10,589	81	0.65	0.63–0.66	0.66	0.65–0.68	0.68	0.63–0.74

Note: Incidence rate is calculated per 100,000 person-years at risk. HR is the estimated hazard ratio per 1 standard deviation improvement in school achievement. Model 1 adjusted for sex, the year of birth, and time-varying calendar year period. Model 2 adjusted for sex, the year of birth, time-varying calendar year period, urbanicity, parental education, parental income, and parental mental health history. Model 3 adjusted for all covariates adjusted in Model 2 but was estimated within strata of full-sibling sets. Mental disorders included: any mental disorders (ICD-10 diagnoses F00–F99), mental and behavioral disorders due to psychoactive substance use (F10–F19), schizophrenia spectrum disorders including schizophrenia, schizotypal, and delusional disorders (F20–F29), bipolar disorder (F30–F31), depression (F32–F33), neurotic, stress-related, and somatoform disorders (F40–F48), eating disorders (F50–F50.9), nonorganic sleep disorders (F51), and disorders of adult personality and behavior (F60–F69).

**Fig. 1:**
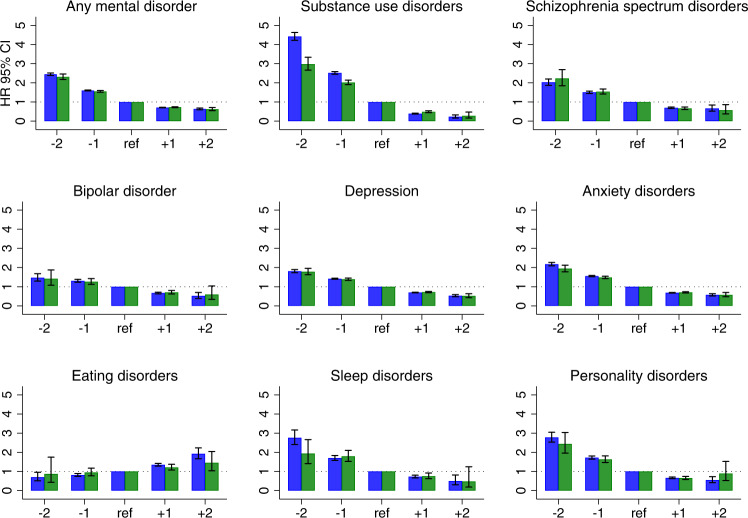
Risk of mental disorders across levels of standardized school achievement in adolescence. Note: The graph displays the results from Model 2 (adjusted for all covariates) in blue and of Model 3 (sibling analyses adjusted for all covariates) in green. The x-axis displays the levels of standardized school achievement. The mean school grades were standardized as z-scores (mean 0, SD 1), and a five-level scale is used: −2SD (≤−2SD); −1SD (>−2SD – ≤−1SD); 0 (>−1SD – <1SD [reference]); 1SD (≥1SD – <2SD); 2SD (≥2SD). The y-axis shows the hazard ratios (HR) and 95% confidence intervals (CI), with average school achievement as the reference group.

A higher level of school achievement was associated with a smaller risk of all subsequent mental disorders, except for eating disorders (Fig. 1). Compared with the average level of school achievement, the poorest level of school achievement (−2SD) was associated with a 2.4-fold risk (HR = 2.45, 95% CI 2.38–2.51) of any subsequent mental disorder, and the highest level of school achievement (+2SD) with almost half the risk (HR = 0.64, 95% CI = 0.60–0.68). For most of the specific psychiatric outcomes, the direction and magnitude of the associations were similar. However, the gradient of the hazard ratios was steeper in the case of substance use disorders, ranging from a 4.4-fold risk (HR = 4.42, 95% CI = 4.22–4.63) with poor school achievement (−2SD) to a very low risk (HR = 0.23, 95% CI = 0.16–0.32) with the poorest level of school achievement (−2SD). Inversely, better school achievement was associated with an increased risk of eating disorders, as the highest level of school achievement (+2 SD) was associated with almost a 2-fold risk (HR = 1.92, 95% CI = 1.66–2.23) and the poorest level of school achievement (−2SD) with approximately only half the risk (−2SD) (HR = 0.70, 95% CI = 0.51–0.96), compared with the risk of a subsequent eating disorder with average school achievement.

In the sibling analyses (Fig. 1), the associations with some specific disorders were diluted (e.g., eating disorders), but most associations remained intact. A notable exception was eating disorders, where poor and below-average school achievement was no longer associated with a smaller risk of an eating disorder diagnosis compared to the risk with average school achievement.

**Fig. 2:**
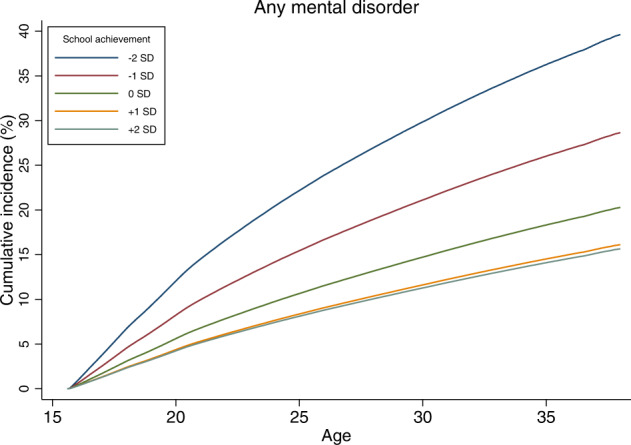
Estimated cumulative incidence of any mental disorder (%) between ages 16 and 38 across levels of standardized school achievement at age 16. The mean school grades were standardized as z-scores (mean 0, SD 1), and a five-level scale is used: −2SD (≤−2SD); −1SD (>−2SD – ≤−1SD); 0 (>−1SD – <1SD); 1SD (≥1SD – <2SD); 2SD (≥2SD).

The absolute risks of developing any subsequent mental disorder associated with the level of school achievement in adolescence, shown as cumulative incidence percentage values, are reported in Fig. 2. The cumulative incidences of any subsequent mental disorder between the ages 16 and 38 were from 39.6% to 28.7% among those with below-average school achievement (−2SD to −1SD), 20.3% with average school achievement, and 16.1% to 15.7% with above-average school achievement (+1SD to +2SD). The cumulative incidences of every mental disorder were smaller at higher levels of school achievement, except for eating disorders, where inversely, the cumulative incidence rates were higher at higher levels of school achievement. The estimated cumulative incidence percentage values for specific mental disorders according to the level of school achievement are reported in Table 2.

**Table 2:** Estimated cumulative incidence as proportions (%) of any mental disorders by the age of 38 years, according to the level of standardized school achievement in adolescence.

Outcome	2SD	−1SD	0	+1SD	+2SD
Any mental disorder	39.6	28.7	20.3	16.1	15.7
Substance use disorders	16.9	9.3	3.4	1.2	0.7
Schizophrenia spectrum disorders	4.8	3.3	2.1	1.4	1.3
Bipolar disorder	1.9	1.8	1.5	1.2	1.0
Depression	15.3	12.8	10.3	8.3	6.9
Anxiety disorders	19.2	14.6	10.6	8.1	7.4
Eating disorders	0.2	0.4	0.8	1.7	3.1
Sleep disorders	2.1	1.3	0.8	0.6	0.4
Personality disorders	4.0	2.5	1.6	1.2	1.0

**Fig. 3:**
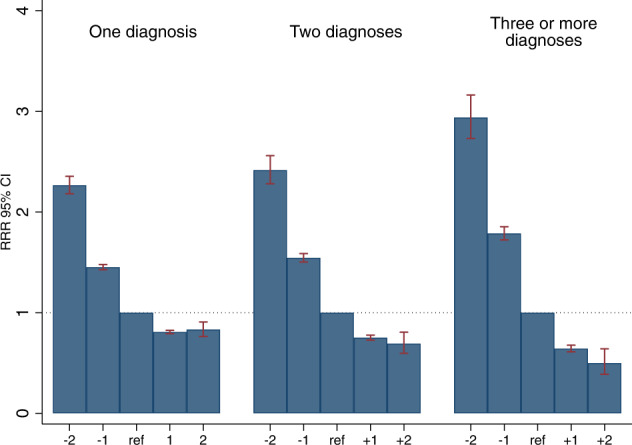
Relative risk ratio (RRR) of comorbid mental diagnoses between ages 16 and 38 across levels of standardized school achievement at age 16. The mean school grades were standardized as z-scores (mean 0, SD 1), and a five-level scale is used: −2SD (≤−2SD); −1SD (>−2SD – ≤−1SD); 0 (>−1SD – <1SD [reference]); 1SD (≥1SD – <2SD); 2SD (≥2SD). Note: The graph displays the results of Model 2 (adjusted for all covariates).

Figure 3 presents the relative risk ratios (RRRs) for the associations of school achievement with psychiatric comorbidity. The associations followed a clear dose-response gradient, with higher school grades associated with a smaller risk of comorbid psychiatric disorders. As shown in Fig. 3, the steepest gradient was observed when the outcome was three or more comorbid diagnoses.

## Discussion

We analyzed nationwide administrative data covering over 1.1 million individuals from Finland who were followed from adolescence up to the age of 38. We observed a graded association between school achievement in the final year of compulsory education (around age 16) and the risk of a subsequent mental disorder across the spectrum of different mental disorders. Poor school achievement was associated with approximately a 2.5-fold risk of any subsequent mental disorder, compared with the risk associated with average school achievement. Moreover, there was a dose-response gradient in the association between the risk and the level of school achievement, with the smallest risk observed at the highest level of school achievement and the association being stronger in lower than in the higher school achievement range. The association was of similar magnitude for all specific mental disorders examined, except for substance use disorders and eating disorder. With eating disorders, the association was of similar magnitude but in the opposite direction, such that better school achievement was associated with a greater risk of a subsequent eating disorder. For substance use disorders, the risk associated with poor school achievement was notably large, over 4-fold compared to the risk associated with average school achievement, and much lower for those with high school achievement. A similar graded association was also observed between school achievement and the risk of psychiatric comorbidity. Compared with the average level of school achievement, poor school achievement was associated with a 3-fold risk of comorbidity, whereas the associated risk was approximately a half with the highest level of school achievement. All these observed associations were robust to adjustment for parental mental disorders, childhood socioeconomic factors, level of urbanicity in residential location, and unobserved shared family characteristics that could affect both school achievement and the incidence of mental disorders.

In terms of absolute risk, the estimated cumulative incidence of any mental disorder was 39.8% at the poorest level of school achievement, 20.3% at the average level of school achievement, and 15.7% at the highest level of school achievement. The magnitude of the association was especially large between school achievement and substance use disorders; at the poorest level of school achievement the cumulative incidence of a later substance use disorder was 17.0%, whereas the corresponding figure at the highest level school achievement was only 0.7%. By contrast, higher school achievement was associated with a greater risk of eating disorders; the cumulative incidence was only 0.2% among those with poor school achievement, 0.8% with average school achievement, and 3.1% with highest school achievement. The difference in the absolute risk between persons at the highest and second highest level of school achievement was often small, suggesting that the association between school achievement and mental disorders was stronger in lower than in the higher school achievement range.

These findings are mostly in accordance with previous epidemiological studies linking poor school achievement to a greater risk of mental disorders [6–12, 14, 22]. A Swedish national register study used Cox regression models to estimate the risk of 11 major mental disorders associated with school achievement [5] measured as grades at the end of compulsory school (~age 16). The HRs related to specific mental disorders were all in the same direction, and only modest differences emerged between our results and those of the Swedish study. Similar results have been reported in data from the UK, where Rahman et al. [10] used linked records measuring school achievement at the age of 7 and 11 years to evaluate associations with subsequent depression and self-harm before the age of 20. They found that those who later self-harmed had a severe decline in their attainment during secondary school, with no difference in their academic attainment in primary school.

In the present study, the association between high school achievement and greater risk of eating disorders was diluted, although it remained, in the sibling analyses, and the same association was fully explained by unmeasured shared family and genetic factors in a Swedish register-based study [23]. There are several plausible explanations for this finding. Previous studies have identified perfectionism, which is a multidimensional personality characteristic, as an important predecessor of both disordered eating behavior [24] and higher school achievement [25]. Thus, perfectionism may partially explain the association between higher school achievement and greater risk of eating disorders. Also, the association between higher childhood socioeconomic status and school achievement has been well established [26], and contrary to other mental disorders [17], higher childhood socioeconomic background has been associated with a greater risk of eating disorders [27]. Moreover, genome-wide association studies have documented a positive genetic correlation between socioeconomic factors and eating disorders [28], suggesting that also these shared genetic factors may contribute to explaining the association between higher school achievement and a greater risk of eating disorders.

Our results further show that poor school achievement is linked to a greater risk of comorbid mental disorders, which has not been documented in earlier literature. The finding is important as psychiatric comorbidity is associated with worse prognosis and outcomes [29] comorbidity likely contributes to the accumulation of various risk factors (within subgroups of people), reinforcing negative consequences for (health and non-health) life outcomes.

Several plausible mechanisms can potentially explain our findings. First, intelligence has a strong association with school achievement [30], lower intelligence in childhood and adolescence has been associated with a higher risk of developing a mental disorder later in life [31–33], and recent evidence suggests that there are overlapping genetic associations across intelligence, school achievement, and mental disorders [34]. Second, school achievement may have causal links to social, educational and economic positions in life, as well as to the development of psychological resiliency, such as self-efficacy or adaptive coping, which can have cumulative adverse or protective effects on mental health. Third, it is likely that some of the mechanisms are mental disorder-specific. For example, a Swedish study using family structure analysis suggested that the association between poor school achievement and the high risk of substance use is due to a combination of lower intelligence, lower school achievement, and family background [12]. Previous literature, in turn, links childhood and adolescent externalizing problems to both poor school achievement and later mental health problems, especially anxiety and depression [11, 35, 36]. Thus, school achievement may indicate risk or protective factors, but the specific mechanisms may vary according to different disorders.

Although the aim of the present study was to examine the association of school achievement at the end of compulsory schooling with subsequent mental disorders, it is important to note that the opposite direction of association, i.e., mental disorders in childhood and adolescence associated with poor school performance, is also relevant. Register-based studies from Denmark and Norway have shown that almost all mental disorders in childhood or adolescence are associated with a lower mean level of school grades [37, 38]. To mitigate potential for reverse causation, we excluded persons with mental disorder diagnoses in childhood and adolescence prior to the follow-up period. However, the first symptoms of later mental disorders often emerge in early adolescence [4]. The possibility that prodromal undiagnosed symptoms negatively affected school performance also among persons included in our study needs to be considered when interpreting our findings.

The main strength of this study was the use of objectively measured longitudinal administrative data (where diagnoses were given by healthcare professionals and the data covered the entire Finnish population), allowing us to account for major socioeconomic and intergenerational confounders, as well as unobserved shared family characteristics that can affect both school achievement and the subsequent incidence of mental disorders. Our empirical approach also has some limitations. First, we excluded persons with mental disorder diagnoses prior to the follow-up period. While this is a standard procedure to reduce reverse causation, it may also induce some selection bias that could affect the estimates. Second, we cannot completely rule out the possibility of unmeasured or residual confounding by non-shared factors or genetics (genetic concordance in full siblings is ~50%), and thus our findings do not have a causal interpretation. Third, outpatient visits in special healthcare were recorded from 1998 onwards and thus it is possible that the first diagnosis of a mental disorder for the oldest birth-year cohorts were not observed. Fourth, the assessment of comorbidity was based on the number of different diagnoses a person received during the follow-up period. Differential diagnosis in psychiatry is complex, and it is possible that some identified instances of comorbidity reflected an arrival at the correct diagnosis after a preliminary diagnosis rather than the onset of a new mental disorder. Finally, the data were from a single Nordic country (i.e., Finland), which has health care system that provides universal access to health care services for all citizens, without notable financial barriers. Consequently, the present findings might not be generalizable to other developed countries with different institutional settings.

## Conclusion

Using accurate and intergenerational educational and mental health data covering 1.1 million individuals from Finland, we observed a dose-response association between the level of school achievement and all major mental disorders as well as psychiatric comorbidity. Poorer school achievement was associated with a greater risk of all other serious mental disorders, except for eating disorders. These findings suggest that the highest mental health burden accumulates among those with the poorest school achievement in adolescence. Further studies are needed to elucidate the mechanisms underlying these findings, and assess the relevance of these findings to mental health promotion.

### Supplementary information

Online Supplement
