# Supplementary material for: School achievement in adolescence and the risk of mental disorders in early adulthood: a Finnish nationwide register study
Source: Mol Psychiatry. 2023 May 2;28(7):3104–10. doi: 10.1038/s41380-023-02081-4 (PMC10615737; doi:10.1038/s41380-023-02081-4)
Supplement: Supplementary file 1 — Online Supplement [file 41380_2023_2081_MOESM1_ESM.docx]

**ONLINE SUPPLEMENT**

**Supplementary Table S1.** Descriptive information of covariates for persons without and with any mental disorder.

|  | **Any mental disorder** | | | |
| --- | --- | --- | --- | --- |
|  | no | | yes | |
| **Sex** | N | % | N | % |
| male | 481159 | 51.9 % | 60983 | 42.3 % |
| female | 445397 | 48.1 % | 83341 | 57.7 % |
| **Mother’s education** |  |  |  |  |
| unknown | 21186 | 2.3 % | 4173 | 2.9 % |
| comprehensive | 128528 | 13.9 % | 27349 | 18.9 % |
| upper secondary | 392750 | 42.4 % | 63101 | 43.7 % |
| lower tertiary | 222339 | 24.0 % | 29352 | 20.3 % |
| Bachelor’s or equivalent | 69593 | 7.5 % | 9213 | 6.4 % |
| Master’s or higher | 92160 | 9.9 % | 11136 | 7.7 % |
| **Father’s education** |  |  |  |  |
| unknown | 41844 | 4.5 % | 9095 | 6.3 % |
| comprehensive | 186104 | 20.1 % | 34741 | 24.1 % |
| upper secondary | 393010 | 42.4 % | 60262 | 41.8 % |
| lower tertiary | 134014 | 14.5 % | 17871 | 12.4 % |
| Bachelor’s or equivalent | 74402 | 8.0 % | 10054 | 7.0 % |
| Master’s or higher | 97182 | 10.5 % | 12301 | 8.5 % |
| **Mother’s income** |  |  |  |  |
| unknown | 21469 | 2.3 % | 4237 | 2.9 % |
| 1st quintile | 171257 | 18.5 % | 37779 | 26.2 % |
| 2nd quintile | 177775 | 19.2 % | 31259 | 21.7 % |
| 3rd quintile | 181387 | 19.6 % | 27649 | 19.2 % |
| 4th quintile | 185600 | 20.0 % | 23434 | 16.2 % |
| 5th quintile | 189068 | 20.4 % | 19966 | 13.8 % |
| **Father’s income** |  |  |  |  |
| unknown | 42959 | 4.6 % | 9372 | 6.5 % |
| 1st quintile | 167490 | 18.1 % | 36227 | 25.1 % |
| 2nd quintile | 173975 | 18.8 % | 29732 | 20.6 % |
| 3rd quintile | 177650 | 19.2 % | 26056 | 18.1 % |
| 4th quintile | 180336 | 19.5 % | 23374 | 16.2 % |
| 5th quintile | 184146 | 19.9 % | 19563 | 13.6 % |
| **Mother’s mental disorder** |  |  |  |  |
| no | 864394 | 93.3 % | 128540 | 89.1 % |
| yes | 62162 | 6.7 % | 15784 | 10.9 % |
| **Father’s mental disorder** |  |  |  |  |
| no | 861048 | 92.9 % | 127752 | 88.5 % |
| yes | 65508 | 7.1 % | 16572 | 11.5 % |
| **Urbanicity** |  |  |  |  |
| unknown | 4133 | 0.4 % | 1859 | 1.3 % |
| urban | 471385 | 50.9 % | 80157 | 55.5 % |
| semi-urban | 177237 | 19.1 % | 25707 | 17.8 % |
| rural | 273801 | 29.6 % | 36601 | 25.4 % |

**Supplementary Table S2.** The association between standardized school achievement in adolescence with later mental disorders for males and females.

|  | Males | | Females | |
| --- | --- | --- | --- | --- |
|  | Model 1 | Model 2 | Model 1 | Model 2 |
| Mental disorder | HR (95% CI) | HR (95% CI) | HR (95% CI) | HR (95% CI) |
| Any | 0.68 (0.67 to 0.68) | 0.68 (0.68 to 0.69) | 0.72 (0.71 to 0.72) | 0.74 (0.73 to 0.74) |
| Substance use | 0.48 (0.47 to 0.49) | 0.49 (0.48 to 0.50) | 0.47 (0.46 to 0.48) | 0.49 (0.48 to 0.51) |
| Schizophrenia spectrum | 0.76 (0.74 to 0.77) | 0.74 (0.72 to 0.75) | 0.73 (0.71 to 0.75) | 0.73 (0.71 to 0.75) |
| Bipolar | 0.86 (0.83 to 0.89) | 0.84 (0.80 to 0.87) | 0.73 (0.71 to 0.75) | 0.75 (0.73 to 0.77) |
| Depression | 0.76 (0.75 to 0.77) | 0.76 (0.75 to 0.77) | 0.73 (0.72 to 0.73) | 0.75 (0.75 to 0.76) |
| Anxiety | 0.69 (0.68 to 0.70) | 0.70 (0.69 to 0.71) | 0.71 (0.70 to 0.72) | 0.73 (0.72 to 0.74) |
| Eating | 1.27 (1.14 to 1.41) | 1.30 (1.16 to 1.46) | 1.33 (1.29 to 1.36) | 1.26 (1.22 to 1.30) |
| Sleep | 0.63 (0.60 to 0.66) | 0.64 (0.61 to 0.67) | 0.72 (0.69 to 0.75) | 0.73 (0.70 to 0.77) |
| Personality | 0.67 (0.65 to 0.69) | 0.69 (0.66 to 0.71) | 0.64 (0.62 to 0.66) | 0.66 (0.64 to 0.68) |

Note: Values are hazard ratios (HRs) with 95% confidence intervals (CIs). Model 1 adjusted for year of birth and time-varying calendar year period. Model 2 adjusted for year of birth, time-varying calendar year period, urbanicity, parental education, parental income, and parental mental health history. Mental disorders included: any mental disorders (ICD-10 diagnoses F00-F99), mental and behavioral disorders due to psychoactive substance use (F10-F19), schizophrenia spectrum disorders including schizophrenia, schizotypal, and delusional disorders (F20-F29), bipolar disorder (F30-F31), depression (F32-F33), neurotic, stress-related, and somatoform disorders (F40-F48), eating disorders (F50-F50.9), nonorganic sleep disorders (F51), and disorders of adult personality and behavior (F60-F69).
